# The use of controlled human infection models to identify correlates of protection for invasive *Salmonella* vaccines

**DOI:** 10.3389/fimmu.2024.1457785

**Published:** 2024-08-27

**Authors:** Naina McCann, Margarete Paganotti Vicentine, Young Chan Kim, Andrew J. Pollard

**Affiliations:** ^1^ Oxford Vaccine Group, Department of Paediatrics, Centre for Clinical Vaccinology and Tropical Medicine, Churchill Hospital, Oxford, United Kingdom; ^2^ NIHR Oxford Biomedical Research Centre, Oxford, United Kingdom

**Keywords:** *Salmonella Typhi* (S Typhi), enteric fever (EF), human challenge model, correlate of protection, vaccine, immunogenicity, *Salmonella*, controlled human infection model

## Abstract

Controlled human infection model (CHIM) studies, which involve deliberate exposure of healthy human volunteers to an infectious agent, are recognised as important tools to advance vaccine development. These studies not only facilitate estimates of vaccine efficacy, but also offer an experimental approach to study disease pathogenesis and profile vaccine immunogenicity in a controlled environment, allowing correlation with clinical outcomes. Consequently, the data from CHIMs can be used to identify immunological correlates of protection (CoP), which can help accelerate vaccine development. In the case of invasive *Salmonella* infections, vaccination offers a potential instrument to prevent disease. Invasive *Salmonella* disease, caused by the enteric fever pathogens *Salmonella enterica* serovar Typhi (*S.* Typhi) and *S.* Paratyphi A, B and C, and nontyphoidal *Salmonella* (iNTS), remains a significant cause of mortality and morbidity in low- and middle-income countries, resulting in over 200,000 deaths and the loss of 15 million DALYs annually. CHIM studies have contributed to the understanding of *S*. Typhi infection and provided invaluable insight into the development of vaccines and CoP following vaccination against *S.* Typhi. However, CoP are less well understood for *S*. Paratyphi A and iNTS. This brief review focuses on the contribution of vaccine-CHIM trials to our understanding of the immune mechanisms associated with protection following vaccines against invasive *Salmonella* pathogens, particularly in relation to CoP.

## Introduction

Enteric fever, caused by *Salmonella enterica* serovar Typhi (*S.* Typhi) and *S.* Paratyphi A, B and C, and invasive nontyphoidal *Salmonella* (iNTS) together cause over 200,000 deaths annually, predominantly among children in low and middle-income countries where many still lack access to clean water and adequate sanitation (1, 2). Although caused by numerous different pathogens with different mechanisms of pathogenesis because of the similar clinical manifestations they will be addressed together in this article. Vaccination offers a possible mechanism to control these diseases, but there have been many barriers to the development of effective vaccines against these pathogens including the lack of an appropriate animal model), the absence of known immunological correlates of protection (CoP) and the need for large numbers of participants for field vaccine efficacy trials (3).

The use of controlled human infection model (CHIM) studies offers a potential solution to some of these barriers, as it allows for vaccine efficacy assessment in a small number of individuals, enabling in-depth investigation of the immune response to vaccination and infection, and, critically, correlation of this response with clinical outcome. This enables investigation of CoP. CoP are immune functions that are statistically correlated with protection from infection. Identification of measurable CoP, such as antibody levels, can aid vaccine design and facilitate early testing of vaccine candidates without the need for large field trials, therefore accelerating vaccine development. The antigens which are thought to be most likely to induce a protective immune response for invasive Salmonella pathogens are the serovar-specific O-antigens (the repetitive glycan polymers of the lipopolysaccharide, LPS), H-antigen (or flagella) and Vi-antigen (present on *S.* Typhi and *S.* Paratyphi C).

## Use of CHIMs to test vaccine efficacy against invasive *Salmonella* pathogens

The first reported use of an invasive *Salmonella* challenge to evaluate a vaccine was in 1896 when Dr Almroth Wright of the Royal Army Medical Corps vaccinated an army officer with killed typhoid bacilli and subsequently challenged him with live *S.* Typhi and found that he did not develop disease (4). However, it was not until 1952 that a reproducible CHIM of *S*. Typhi was established at the University of Maryland. Using this model, over 1,800 participants were challenged with *S.* Typhi over a period of 20 years, and 10 different *S.* Typhi vaccine candidates were evaluated (see **Table 1**) until 1974, when this model was discontinued.

**Table 1:** Summary of CHIM vaccine-challenge studies of invasive *Salmonella*.

Site	Dates	Vaccine candidate	Vaccine dosing	Challenge Pathogen	Efficacy	References
University of Maryland	1967	Vaccine K (parenteral acetone-inactivated) (n= 28 vaccine, n = 30 control)	3 doses at 0,14 and 35 days	*S.* Typhi Quailes strain given 3m – 12m after last vaccine	No protection with challenge dose of 10^6^ CFU *	Hornick 1967 (8)
University of Maryland	1967	Vaccine K (parenteral acetone-inactivated) (n = 43 vaccine, n = 104 control)	3 doses at 0,14 and 35 days	*S.* Typhi Quailes strain given 3m – 12m after last vaccine	67% efficacy with challenge dose of 10^5^ CFU *	Hornick 1967 (8)
University of Maryland	1967	Vaccine L (parenteral phenol-heat inactivated) (n = 24 vaccine, n = 30 control)	3 doses at 0,14 and 35 days	*S.* Typhi Quailes strain given 3m – 12m after last vaccine	No protection with challenge dose of 10^6^ CFU *	Hornick 1967 (8)
University of Maryland	1967	Vaccine L (parenteral phenol-heat inactivated) (n = 45 vaccine, n = 104 control)	3 doses at 0,14 and 35 days	*S.* Typhi Quailes strain given 3m – 12m after last vaccine	67% efficacy with challenge dose of 10^5^ CFU *	Hornick 1967 (8)
University of Maryland	1970	Parenteral denatured Vi polysaccharide (n= 17)	Single dose	*S.* Typhi Quailes strain given 3m – 12m after last vaccine	Not protective *	Hornick 1970 (9)
University of Maryland	1971	Taboral tablets (monovalent preparation containing acetone-killed *S*. typhosa strain Ty2) (n= 72)	2 tablets a day for 3 days (n = 43) 4 tablets a day for 3 days (n = 29)	*S.* Typhi Quailes strain given 8-10 weeks after vaccination	Not protective *	DuPont 1971 (10)
University of Maryland	1970	Freshly harvested attenuated streptomycin dependent oral vaccine (n= 45)	3 x 10^10-11^ weekly for 5 weeks. Oral streptomycin given with last 2 doses.	*S.* Typhi Quailes strain given 6 weeks after completing vaccination	66% efficacy *	Levine 1976 (11)
University of Maryland	1972	Freshly harvested attenuated streptomycin dependent oral vaccine (n = 44)	3 x 10^10^ twice a week for 4 weeks (between 5-8 doses)	*S.* Typhi Quailes strain given 6 weeks after completing vaccination	72% efficacy *	Levine 1976 (11)
University of Maryland	1972	Lyophilised attenuated streptomycin-dependent oral vaccine (n= 62)	5 x 10^10^ weekly for 4 weeks. Streptomycin with each dose	*S.* Typhi Quailes strain given 6 weeks after completing vaccination	Not protective *	Levine 1976 (11)
University of Maryland	1973	Lyophilised attenuated streptomycin-dependent oral vaccine. (n = 25)	3 x 10^10^ (first dose), 4 x 10^10^ (2^nd^ dose), 5 x10^10^ (3^rd^ to 8^th^ dose). No streptomycin. Two doses per week	*S.* Typhi Quailes strain given 6 weeks after completing vaccination	Not protective *	Levine 1976 (11)
University of Maryland	1977	Ty21a (galE mutant of *S*. Typhi) grown with exogenous 0.1% galactose. (n = 99)	5-8 oral doses at 3-4 day intervals over 4 weeks	*S.* Typhi Quailes strain given 5-9 weeks after completing vaccination	87% efficacy *	Gilman 1977 (12)
University of Maryland	1977	Ty21a (galE mutant of *S.* Typhi) grown without exogenous galactose. (n = 56)	5-8 oral doses at 3-4 day intervals over 4 weeks	*S.* Typhi Quailes strain given 5-9 weeks after completing vaccination	Not protective *	Gilman 1977 (12)
University of Oxford	2011-2012	M01ZH09 (n= 31), Ty21a (n = 30) Placebo (n = 30)	M01ZH09 = 1 oral dose, Ty21a = 3 oral doses, Placebo = 1 oral dose	*S.* Typhi Quailes strain given 4 weeks after vaccination	M01ZH09 = 13% ([95% CI -29 to 41] not significant) Ty21a = 35% ([95% CI -5 to 60] not significant)	Darton 2016 (13)
University of Oxford	2014	Vi-TT (n =37), Vi-PS (n = 35), Control (n = 31)	All 1 intra-muscular dose	*S.* Typhi Quailes strain given 4 weeks after vaccination	Vi-TT = 54% (95% CI 26.8 to 71.8) Vi-PS = 52% (95% CI 23.2 to 70)	Jin 2017 (14)
University of Oxford	2022-2024	CVD 1902 Placebo	2 doses 14 days apart	*S.* Paratyphi A strain NVGH308 given 4 weeks after vaccination	Trial ongoing	McCann (6)

*Confidence interval not available.

In 2009, a modern-day *S.* Typhi CHIM was established at the University of Oxford. Approximately 350 participants have now been challenged with *S.* Typhi in this CHIM, with four *S*. Typhi vaccine candidates evaluated (see **Table 1**). This has provided further insights into potential CoP against infection by this pathogen. In 2014 we also established a *S*. Paratyphi A CHIM, and the first CHIM study to evaluate a *S.* Paratyphi A vaccine is currently ongoing (5, 6). Additionally, investigators at Imperial College London are in the process of establishing the first iNTS CHIM (7).

**Figure 1:**
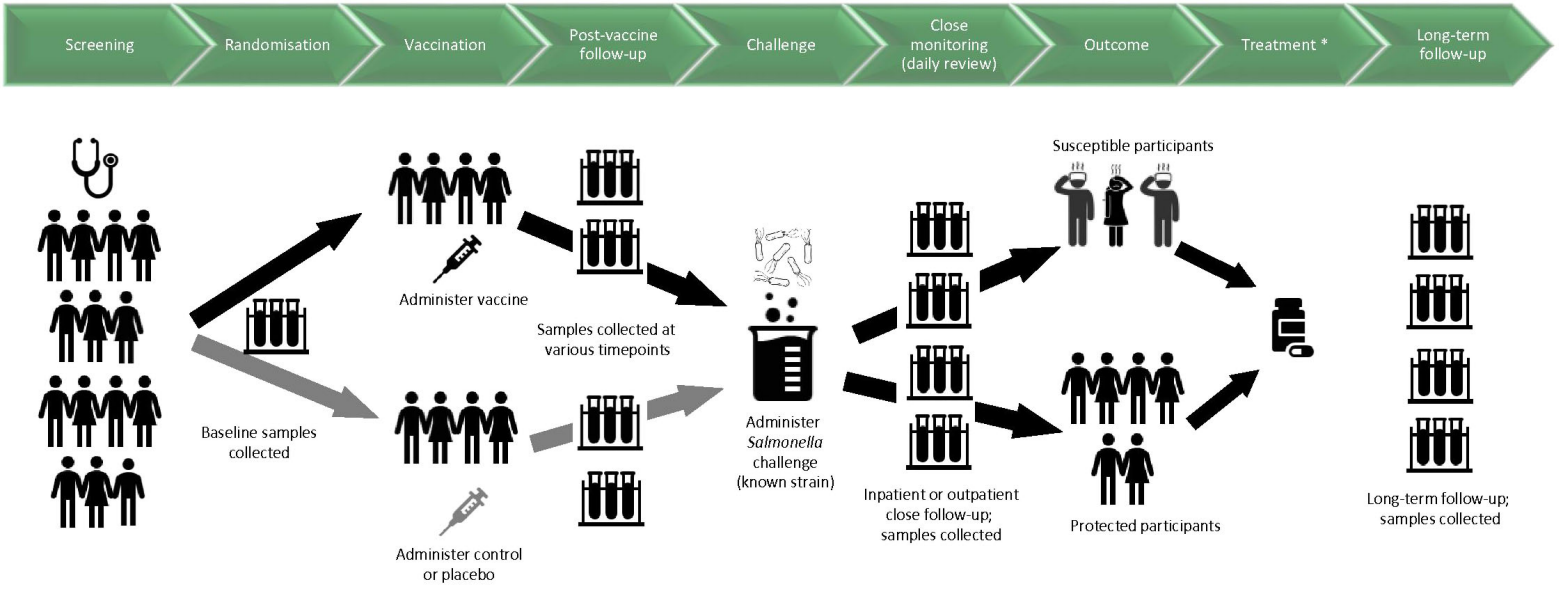
Schematic diagram for timelines and procedures in a CHIM investigating vaccines against invasive disease caused by Salmonella. Samples relate to blood, saliva, urine, faeces and/or any other samples that may be involved in the study. Challenge was given 3-12 months after vaccination at the first CHIM trials at the University of Maryland; for later CHIM trials at the University of Maryland and at the University of Oxford, challenge is given 4-8 weeks after vaccination. Participants are considered susceptible if they are diagnosed according to predetermined criteria. *The antimicrobial treatment used in early CHIMs conducted at the University of Maryland was not given to all participants as in later CHIMs, but to susceptible participants according to criteria for diagnosis.

**Figure 1** shows a schematic for the timelines and procedures typically involved for a vaccine-CHIM.

## Inactivated whole cell vaccines

Inactivated whole cell vaccines against *S*. Typhi (either acetone-killed or heat-inactivated) were developed at the end of the 19th century and widely used in military personnel during World War, however they are no longer available due to high reactogenicity. These vaccines were first evaluated in the typhoid CHIM at the University of Maryland in the 1960s (8). No protective efficacy following vaccination was seen at a challenge dose of 10^6^ bacteria, but when this dose was lowered to 10^5^, vaccine efficacy was 67% (8). Antibody titres to O, H and Vi antigens, presumed the most likely protective antibodies, were measured, but there was no correlation between pre-challenge antibody titres and protection from illness (8, 15, 16). This finding was replicated in further CHIM studies at the University of Maryland evaluating a killed oral vaccine (Taboral), which did not protect from infection nor induce a significant antibody serological response to O, H or Vi antigens in the majority of participants (10). DuPont et al. found no correlation between aa 4-fold rise in serum agglutinins following oral vaccination with the killed oral vaccine typhoid fever development following *S*. Typhi challenge (10).

## Live attenuated vaccines

The Maryland team conducted four trials of an oral live attenuated streptomycin-dependent *S.* Typhi vaccine [attenuated to be streptomycin-dependent by repeated cultivation of a pathogenic *S.* Typhi strain in the presence of streptomycin (17)]: two using freshly harvested bacteria and two using lyophilised bacteria in the vaccine (11, 18). The vaccine containing freshly harvested bacteria (grown in culture and then directly administered) were more efficacious (66% and 72% efficacy for freshly harvested vaccine compared to 0% and 28% for the lyophilised vaccine) and induced more participants to develop a four-fold rise in circulating anti-H titre from baseline (58% vs 33%), compared with the lyophilised vaccine. As the methodology for antibody detection was the same for all studies this difference is most likely explained by differences in the vaccines; lyophilisation appears to reduce both efficacy and immunogenicity of the vaccine and therefore must affect these streptomycin-dependent organisms in some way. Antibody response to O-antigen was rarely observed and there was no correlation between presence of O or Vi-antibody and protection against clinical disease (11). However, it was found that unvaccinated participants with high baseline anti-H titres had a lower attack rate than unvaccinated participants without high anti-H titres (22% vs 48%, p = 0.005), although this effect was not seen in vaccinees.

Another CHIM study evaluated a different live attenuated vaccine, Ty21a, a galE mutant of the Ty2 wild-type *S.* Typhi strain, grown either with or without galactose (12). Only the vaccine using bacteria grown with galactose was protective from infection, and it induced a higher rate of O-antigen seroconversion (a = fourfold rise in antibody from baseline) than the vaccine containing bacteria grown without galactose (17% vs 4%, p <0.04), but this did not correlate with protection from infection Rates of seroconversion to H-antigen were higher in both groups (60% in galactose negative and 60%in galactose positive) but there was minimal seroconversion to Vi-antigen (2% in galactose negative and 0% in galactose positive). In vaccinees, there was no correlation between presence of H or Vi antibodies and protection from infection. In concordance with the study of streptomycin-attenuated vaccines discussed above (11), unvaccinated volunteers who had pre-existing detectable titres of antibodies to H antigen had lower attack rates than unvaccinated volunteers without detectable H antibodies (24% vs 61%, p =0.02). This effect was not seen in vaccinees but suggests a possible protective role for pre-existing H-antigen.

In 2011, Ty21a and another live attenuated vaccine, M01ZH09, were assessed in the *S*. Typhi CHIM study at Oxford University (13). Vaccine efficacy was 13% (95% CI -29 to 41%) for a single dose of M01ZH09 and 35% (95% CI -5 to 60%) for 3 doses of Ty21a. M01ZH09 generated significant increases in geometric mean titres of all anti-LPS and anti-H antibody classes from baseline to 28 days following vaccination when compared to placebo, but no significant increase in anti-Vi IgG titres (13). Ty21a generated a borderline significant increase in anti-LPS IgG only, but when looking at antibody secreting cell titres (as measured by ELISPOT) both vaccines induced a significant increase in anti-LPS ASC and anti-H ASC compared with placebo. However there was no correlation between generation of a humoral antibody response and protection, aligning with data from field trials and suggesting alternative mechanisms of protection (19).

Juel et al. investigated the functionality of antibodies induced by these vaccines and showed that vaccine-derived anti-LPS antibodies following M01ZH09 induced antibody-dependent complement activation and bacterial killing that correlated with delayed disease onset and decreased disease severity, but not protection from infection (20). This suggests that although other immune mechanisms are at play to protect against typhoid disease following vaccination with M01ZH09, bactericidal antibodies may reduce bacterial load in early infection to reduce disease severity. This is similar to findings from a Shigella CHIM study which showed higher pre-challenge SBA titres correlated with decreased disease severity (21).

Using data from the same M01ZH09 and Ty21a Oxford CHIM study (13), Blohmke et al. investigated the transcriptional responses to these two vaccines, finding marked differences between them, despite the two vaccines originating from the same original strain (22). M01ZH09 was associated with significant cell cycle activation which was correlated with anti-O:9:LPS and anti-H antibody responses, whereas Ty21a was associated with NK cell activity (23). Authors postulated that this NK cell activation may be involved in protection at the mucosal level, and previous studies have shown that cellular responses induced by Ty21a are primed for gut homing (24, 25). Authors also found transcriptional signatures of amino acid metabolism in Ty21a recipients were associated with protective outcomes (longer incubation time and lower temperature) and (22). suggested they may be representative of activation of a CD8+ T-cell response, previously shown to be induced by Ty21a (26).

A live attenuated vaccine against *S.* Paratyphi A is currently being evaluated in the *S*. Paratyphi A CHIM in Oxford, with results expected in 2024 (6). Phase 1 data showed this vaccine elicited *S.* Paratyphi A-specific IgG and IgA and cell-mediated responses in individuals, including B memory cells and multifunctional CD8+ T cells expressing the gut homing marker α4β7, suggesting this vaccine may induce a mucosal cell-mediated immune response (27). B memory cells against LPS correlate with protection from infection by other enteric pathogens (e.g., cholera, shigella) and therefore may also play an important role in protection against *S.* Paratyphi A (28, 29). Further investigation of these responses in this *S.* Paratyphi ACHIM, coupled with clinical outcome data, will provide invaluable information on possible CoP.

## Vi capsular polysaccharide-based vaccines: plain and conjugate vaccines

In 2015, a *S*. Typhi CHIM trial was conducted in Oxford to assess the efficacy of a typhoid conjugate vaccine containing Vi polysaccharide conjugated to tetanus toxoid (Vi-TT). The trial compared Vi-TT with the licensed Vi-polysaccharide vaccine (Vi-PS) and a control vaccine (against meningococci) (14). Investigators showed that both typhoid vaccines had similar efficacy (54.6% [95% CI 26.8-71.8] for Vi-TT, 52% [95% CI 23.2-70] for Vi-PS) and induced significant anti-Vi responses compared with baseline. However, Vi-TT induced higher rates of seroconversion (a > four-fold rise in antibody titre 28 days after vaccination) (100% vs 88.6%) and higher geometric mean titres (GMT) (562.9 EU/mL vs 140.5 EU/ml) than Vi-PS. Overall, authors showed that participants with higher anti-Vi IgG titres had lower odds of typhoid diagnosis (14).

The humoral response to Vi-TT and Vi-PS was studied further by Dahora et al. who characterised different antibody isotypes and subclasses and their avidity, and by Jin et al. who used a systems serology approach to study Vi-associated CoP post-vaccination in the same set of participants (30, 31). The main antibody induced by both Vi vaccines was IgA (mainly IgA1), followed by IgG2, at significantly higher response rate and levels in Vi-TT than in Vi-PS vaccine responders. Magnitude of IgA response and fold-change in IgA levels were higher in protected participants and could predict protection in Vi-PS vaccinees (31). When investigated further in Vi-PS vaccinees, authors found Vi IgA2 levels were six-fold higher in protected versus susceptible individuals. Given that IgA2 is found predominantly in the mucosa this suggests a possible protective mechanism for IgA2 at the local gut epithelium following Vi-PS vaccination.

Jin et al. corroborated these findings, showing that protected individuals had higher absolute and fold increases for Vi IgA responses (total Vi IgA titre, IgA subclasses and FcαR binding) than susceptible individuals. A predominant IgA response to Vi vaccination has been shown in previous studies (24, 32) and secretory IgA has been correlated with protection for other enteric pathogens (polio, rotavirus) (33, 34), although there have been limited studies to date investigating the role of IgA in protection following parenteral vaccination. For *S*. Typhi, since the Vi-capsular antigen is only expressed extracellularly for a short period (due to the inhibitory effect of the high osmolality in the gut lumen on Vi expression), it has been proposed that high levels of IgA in the gut mucosa may protect by opsonising the extracellular *S.* Typhi, thus preventing infection (30).

In these two papers, anti-Vi IgG was also found to be significantly higher in protected versus susceptible participants across both vaccine groups, particularly IgG2. IgG2 has previously been shown to correlate with the mean antibody response following exposure to bacterial polysaccharides (35). Jin et al. also showed that fold changes in anti-Vi IgG correlated with improved clinical outcomes such as lower temperatures and bacterial burden (30). Authors therefore suggested a role for Vi IgG in controlling disease severity by acting to reduce bacterial burden in the bloodstream in the early stages of infection, and suggested this may be used as a CoP for typhoid fever disease.

Going on to evaluate functional responses, Jin et al. showed higher fold increases in antibody-dependent neutrophil oxidative burst activity (ADNOB, p = 0.014) and antibody dependent neutrophil phagocytosis (ADNP, p = 0.062) in protected participants following vaccination versus susceptible participants. Further multivariate analyses identified anti-Vi IgA quantity, anti-Vi IgG2 and IgA2 avidity as key protective features, while antibody-dependent NK activation (ADNKA) predicted infection. Authors suggested these results point to a coordinated protective response of selective neutrophil activation mediated by both Vi IgA and IgG.

Diverse humoral immunological signatures of Vi-PS and Vi-TT were identified by both sets of authors. Protection following Vi-PS was found to be polyisotypic (IgA, IgM and IgG) and polyfunctional, aligning with findings from other studies of plain polysaccharide vaccines, such as the pneumococcal vaccine (36). Protection following Vi-TT was found to be primarily correlated with anti-Vi IgA (quantity and avidity), IgG1 avidity and antibody-dependent neutrophil phagocytosis (ADNP). It is known that adding a protein conjugate to a polysaccharide vaccine can increase vaccine response through engagement of T-cells and stimulation of a germinal centre reaction that leads to affinity maturation and memory formation. Prior studies have shown that a tetanus carrier protein induced higher avidity antibodies than other licensed protein carriers (37, 38). Dahora et al. did find the avidity of each antibody subclass was higher in Vi-TT participants than those receiving Vi-PS although, bar IgG3, these differences were not significant.

Antibody glycosylation, which involves modification of the N-linked glycosylation site of the Fc-region of the antibody, can affect antibody stability and effector functions. Stockdale et al. investigated changes in glycosylation profile of total circulating IgG following Vi-TT and Vi-PS in the human challenge study and found vaccination with Vi-TCV induced higher Vi-specific glycan changes of higher magnitude than Vi-PS, although there was no correlation of glycosylation profiles with protection from typhoid fever (39).

Jones et al. studied the serum bactericidal activity (SBA) in samples from these participants (40). They demonstrated that, despite both vaccines (Vi-TT and Vi-PS) eliciting an increase in SBA titres, this was not correlated with protection nor with reduced disease severity. This contrasted with findings from other studies of Gram-negative encapsulated bacteria such as *Neisseria meningitidis* where SBA and/or vaccine-induced opsonophagocytosis has been shown to correlate with protection (41). This also contrasts with findings from a previous study in Nepal showing an age-dependent increase in SBA titres to *S*. Typhi, suggesting a potential relationship between titres and protection from disease (42). While differences in the assay methodologies could be further explored, one proposed reason is that as Vi-antigen is only expressed after translocating to the lamina propria, where low levels of complement are present, this could reduce the observed effect of SBA (40).

Using samples from the same trial, Cross et al. investigated the leucocyte response following vaccination, using mass cytometry and ELISpot assay (43). They found that Vi-PS and Vi-TT both induced antibody-secreting cells at 7 days post-vaccination. Only Vi-TT, as expected, induced a memory B cell response and, to a smaller extent, a non-B cell response (including T follicular cells). Additionally, the Vi-specific IgG and IgM B cell response was of greater magnitude in Vi-TT vaccinees compared with Vi-PS recipients. Interestingly, both vaccines induced the expansion of gut homing B cells, which may play an important role in vaccine-induced protection post either Vi-PS or Vi-TT (44). Importantly, the total plasma cell response was associated with protection in the Vi-TT arm. Results from studies of other enteric pathogens (e.g., *Shigella*) and on experimental *S.* Typhi infection have also demonstrated augmented plasma cell response, both systemic and mucosa-directed (45, 46). Transcriptomic changes in response to vaccination in these samples were analysed by Zhu et al. who found that both vaccines led to upregulation of neutrophil degranulation pathway and plasma cell signals (47). After Vi-TT vaccination, a significantly higher number of overexpressed genes were observed compared with post Vi-PS, including interferon signalling pathway, CD4+ T cells, IgG1 subclass response and multiple immunoglobulin heavy chain variable regions. Investigating the B-cell-receptor (BCR) repertoire of hyperexpanded clonotypes after Vi-TT vaccination, Zhu et al. observed more robust and diverse clonal expansion targeting Vi-antigen in protected versus susceptible participants, indicating a correlation with vaccine-induced protection.

## Limitations of CHIMs to profile *Salmonella* vaccine immunogenicity

There are multiple limitations to using CHIMs to profile *Salmonella* vaccine immunogenicity and CoP. Firstly, the studies are powered to demonstrate efficacy, not CoP, which could reduce the significance of findings. Furthermore, in CHIMs a specific dose of a specific strain (e.g., 10^4^ CFU of Quailes in Oxford trials) is administered after a specific interval following vaccination, whilst in the community, any strain at varying inoculum levels could lead to infection. Thus, if force of infection is lower in the field than in the CHIM, the CHIM may over-estimate the level of immune marker required for protection. Additionally, the population challenged in this model differs from that in endemic settings (genetics, co-morbidities, previous exposure to the agent before vaccination). Notably, the population challenged in CHIMs consists of adults, whereas the disease mostly affects school-age children in endemic countries. Although both groups are expected to be infection-naïve, immune response in these two age-groups may differ, in particular B cell differentiation and affinity maturation in germinal centres and the maintenance of long-lived plasma cells is known to change with age. However, *Salmonella* CHIMs conducted in endemic areas would enable comparison of levels of protective immunity between residents of these areas and naïve populations in high income countries and should be encouraged.

## Discussion and future directions

CHIM studies evaluating *S.* Typhi vaccines have provided valuable information on the immune responses generated by different *S*. Typhi vaccines and led to the identification of vaccine-induced CoP for Vi-vaccines. Efficacy results from the trial comparing Vi-TT and Vi-PS (14) were used to support WHO recommendations, ultimately leading to the administration of over 60 million doses of typhoid conjugate vaccines (TCVs) to safeguard populations from low- and middle-income countries against this disease. The identification of Vi IgA and IgG responses as correlates of protection following Vi vaccination from this same trial has been vital in allowing monitoring of the vaccine response following the TCV implementation and supporting and accelerating the licensure pathway for newly developed TCVs (48, 49).

In contrast, although over 150 million doses of Ty21a have been administered globally the mechanisms by which this vaccine protects against typhoid disease remain poorly understood, with little evidence to support a CoP following live attenuated vaccination. Further work to investigate the local mucosal antibody response, as well as the cellular response following both live attenuated and parenteral conjugated vaccines against invasive *Salmonella* infection in the CHIM may yield more insightful data and should be considered in future studies.

Although significant steps have been made in the fight against typhoid, comprehensive control of the invasive diseases caused by *Salmonella* pathogens requires vaccines against *S.* Paratyphi A and iNTS. With recent advances in immunological and molecular techniques coupled with the ability to collect samples at timepoints close to infection, CHIM studies offer significant potential to enhance our understanding of vaccine-induced CoP for these neglected diseases and accelerate the pathway to vaccine licensure.
